# Co-resistance to Amoxicillin and Tetracycline as an Indicator of Multidrug Resistance in *Escherichia coli* Isolates From Animals

**DOI:** 10.3389/fmicb.2019.02288

**Published:** 2019-10-09

**Authors:** Clémence Bourély, Géraldine Cazeau, Nathalie Jarrige, Eric Jouy, Marisa Haenni, Agnese Lupo, Jean-Yves Madec, Agnès Leblond, Emilie Gay

**Affiliations:** ^1^École Nationale des Services Vétérinaires, ENSV, VetAgro Sup, Marcy l’Étoile, France; ^2^ANSES, Laboratoire de Lyon, Unité Épidémiologie et Appui à la Surveillance, Université de Lyon, Lyon, France; ^3^EPIA, UMR 0346, Epidémiologie des Maladies Animales et Zoonotiques, INRA, VetAgro Sup, University of Lyon, Marcy l’Étoile, France; ^4^Laboratoire de Ploufragan-Plouzané-Niort, ANSES, Unité Mycoplasmologie Bactériologie Antibiorésistance, Université Bretagne Loire, Technopôle Saint-Brieuc Armor, Ploufragan, France; ^5^ANSES, Laboratoire de Lyon, Unité Antibiorésistance et Virulence Bactériennes, Université de Lyon, Lyon, France

**Keywords:** antimicrobial resistance, multidrug resistance, *E. coli*, animal health, RESAPATH

## Abstract

**Objectives:**

To examine the relevance of co-resistance to amoxicillin and tetracycline as an indicator of multidrug resistance (MDR) in animal health.

**Methods:**

*Escherichia coli* isolates collected between 2012 and 2016 by the French surveillance network for antimicrobial resistance in diseased animals (RESAPATH) were analyzed. The proportions of MDR isolates and the proportions of isolates presenting co-resistance to amoxicillin and tetracycline were calculated for seven animal species (cattle, horse, dog, swine, poultry, duck, and turkey). The degree of agreement between these two proportions was estimated by calculating the kappa value.

**Results:**

In total, 55,904 isolates were analyzed. MDR proportions were variable among animal species, ranging from 21.9% [20.2; 23.7] in horses to 56.0% [55.4; 56.7] in cattle. A similar situation was observed for proportions of isolates with co-resistance to amoxicillin and tetracycline, with the highest value for cattle 65.0% [64.3; 65.6]. This co-resistance was also most often associated with resistance to other antibiotics, regardless of the animal species considered. Comparative analysis showed substantial agreement between MDR and this co-resistance, with a kappa value of 0.75, all animal species considered.

**Conclusion:**

Given the widespread use of penicillins and tetracyclines in animal health, co-resistance to amoxicillin and tetracycline could be an efficient indicator of MDR in *E. coli* isolates. Based on a specific resistance profile and not an arbitrary number of resistances compared with MDR, this potential indicator is also precise, convenient and suitable for routine use.

## Introduction

*Escherichia coli* is the most frequently isolated pathogen in food-producing animals and in pets and a major Gram-negative infectious agent for humans, being particularly implicated in urinary tract infections (Karam et al., 2016). *E. coli* is also considered to be an excellent sentinel of antimicrobial resistance for a wide range of animal species (De Graef et al., 2004; Aarestrup et al., 2008). Characterization of resistance in *E. coli* isolates include the assessment of their multidrug resistance (MDR), which should follow a standard definition.

Schwarz et al. (2010) proposed a general definition of MDR in veterinary medicine: MDR is the acquired resistance to at least one antibiotic in three or more antibiotic categories (Schwarz et al., 2010). Because MDR status is based on the number of resistances, it is directly related to the total number of antibiotic categories selected for analysis (Magiorakos et al., 2012). However in the literature, regardless of the bacterial species considered, several definitions are used with no consensus regarding the number of antibiotic categories to take into account or the threshold to define MDR, i.e., the number of acquired resistances. Some authors take into account five acquired resistances (Strand et al., 2014; Aperce et al., 2016), whereas others consider only two (Cummings et al., 2015) or do not even specify which definition they use (Ewers et al., 2014). Therefore, assessment of MDR is inconsistent across studies and the variability of definitions hinders comparing studies (Cohen et al., 2008). To address these limitations, the MDR definition should be supplemented with an indicator describing a specific antibiotic resistance profile, i.e., co-resistance to several antibiotics.

In *E. coli* isolates from animals, resistances to amoxicillin and tetracycline are among the most frequently encountered resistances all over the world (Chen et al., 2014; Hanon et al., 2015; Boireau et al., 2018b; EFSA and ECDC, 2018). Furthermore, penicillins and tetracyclines are the most frequently used antibiotics in veterinary medicine in Europe (EMA and ESVAC, 2018). Therefore, the aim of this study was to examine if co-resistance to amoxicillin and tetracycline can be an indicator of MDR in animal health.

## Materials and Methods

This study was performed using data from the RESAPATH, the French established national surveillance network for antimicrobial resistance (AMR) in bacteria from diseased animals. The RESAPATH collects results from antimicrobial susceptibility testing performed routinely by French veterinary laboratories. Even if each laboratory has its own strategy for bacterial identification, API galleries are often used and the biggest ones use Maldi-TOF. All laboratories perform antimicrobial susceptibility testing by the disk diffusion method following the veterinary recommendations of the Antibiogram Committee of the French Society for Microbiology (CA-SFM)^1^.

From the RESAPATH database, data regarding *E. coli* isolates from 2012 to 2016 were extracted. The seven animal species with the largest amount of data were selected: cattle, horse, dog, swine, poultry (*Gallus gallus*), turkey, and duck. For the analysis, seven antimicrobial categories of relevance in veterinary and human medicine were selected: penicillins, penicillins with beta-lactamase inhibitors, extended-spectrum cephalosporins, aminoglycosides, tetracyclines, folate pathway inhibitors and fluoroquinolones (Table 1). The antibiotics selected were based on antibiotic use in veterinary medicine and on veterinary laboratory practices in France.

**TABLE 1:** Antibiotics selected and relative antimicrobial categories used to define multidrug resistance.

**Antimicrobial category**	**Antibiotic**
Penicillins	Amoxicillin
Penicillins and beta-lactamase inhibitors	Amoxicillin and clavulanic acid
Extended-spectrum cephalosporins	Ceftiofur
Aminoglycosides	Gentamicin
Tetracyclines	Tetracycline
Fluoroquinolones	Enrofloxacin or danofloxacin or marbofloxacin
Folate pathway inhibitors	Trimethoprim-sulfamethoxazole

Isolates with acquired resistance to at least one antibiotic in three or more antibiotic categories selected were considered as multidrug resistant (Schwarz et al., 2010). MDR proportions and the proportion of isolates with co-resistance to amoxicillin and tetracycline were calculated by animal species and confidence intervals (CI) were estimated using the exact binomial method (two sided *p*-values). The degree of agreement between the number of multidrug resistant isolates and the number of isolates presenting co-resistance to amoxicillin and tetracycline was estimated by calculating the kappa value. Kappa (*k*) values of ≥0.81, 0.61–0.80, 0.40–0.60, and ≤0.40 were considered to represent excellent, substantial, moderate to good, and slight to poor agreement, respectively (Viera and Garrett, 2005). R Core Team (2017), R version 3.4.3. (2017-11-30) was used for all statistical analyses.

## Results and Discussion

A total of 55,904 *E. coli* isolates were analyzed for the 2012–2016 period (Table 2). MDR varied among animal species, ranging from 21.9% in horses to 56.0% in cattle. The proportion of isolates with co-resistance to tetracycline and amoxicillin was the highest for cattle (65.0%) followed by swine, ducks, and turkeys. The proportion of isolates with co-resistance to amoxicillin and tetracycline was higher than the proportion of multidrug resistant isolates for all food-production animals, but lower for companion animals (dog and horses). These differences are probably due to variations in antibiotic use among animal species (Anses-ANMV, 2018), despite the widespread use of amoxicillin and tetracycline in domestic animals. These elements suggest that the capacity and relevance of the co-resistance to amoxicillin and tetracycline as a proxy for MDR might be linked to the context of antibiotic use. Similar studies are necessary to confirm these results in area where antibiotic use could be different.

**TABLE 2:** Proportion (%[CI]) of multidrug resistance and of co-resistance to amoxicillin and tetracycline in *E. coli* isolates per animal species, over the 2012–2016 period.

**Animal species**	**Number of isolates**	**Proportion of multidrug resistance**	**Proportion of co-resistance to amoxicillin and tetracycline**
Cattle	21,451	56.0 [55.4; 56.7]	65.0 [64.3; 65.6]
Horse	2,232	21.9 [20.2; 23.7]	17.7 [16.1; 19.3]
Dog	3,226	23.3 [21.9; 24.8]	20.1 [18.8; 21.6]
Swine	5,932	44.9 [43.6; 46.2]	46.7 [45.4; 48.0]
Poultry	16,090	22.3 [21.7; 23.0]	26.0 [25.3; 26.7]
Turkey	4,125	24.8 [23.5; 26.1]	36.5 [35.1; 38.0]
Duck	2,848	39.7 [37.9; 41.6]	45.3 [43.5; 47.2]

Over the whole period, the concordance between the multidrug resistant *E. coli* isolates and the isolates with co-resistance to amoxicillin and tetracycline was substantial with an overall kappa value of 0.75, all animals included. More specifically, the concordance was excellent for horses (*k* = 0.83) and substantial for other animal species (Table 3). Calculated per year and all animals included, the degrees of agreement were always substantial, varying between 0.71 and 0.76 over the period.

**TABLE 3:** Contingency table of multidrug resistance and co-resistance to amoxicillin and tetracycline in *E. coli* isolates from all animals considered, over the 2012–2016 period, with percentages by row (% R) and by column (% C).

**Profile**	**Multidrug resistant isolates**		**Non-multidrug resistant isolates**		**Total**
Isolates with co-resistance to amoxicillin and tetracycline	19,732	79.8% R	4,995	20.2% R	24,727
		91.0% C		14.6% C	
Isolates without co-resistance to amoxicillin and tetracycline	1,945	6.2% R	29,232	93.8% R	31,177
		9.0% C		85.4% C	
Total		21,677		34,227	55,904

Based on the contingency table, only 9.0% (1,945/21,677) of multidrug resistant isolates did not display co-resistance to amoxicillin and tetracycline (Table 3). This co-resistance was most frequently associated with other resistances, regardless of the animal species (Tables 3, 4 and Supplementary Tables S1, S2). In fact, among all animals, 79.8% (19,732/24,727) of isolates with co-resistance to amoxicillin and tetracycline were also multidrug resistant (Table 4) and this proportion ranged from 65.2% for turkeys to 97.0% for horses (Table 3). However, 20.2% (4,995/24,727) of isolates with this co-resistance did not show other resistances and were not multidrug resistant (Table 4). In addition, variability among animals was observed (Table 3). Despite these limitations, our findings highlight that, given the widespread use of penicillins and tetracyclines, co-resistance to amoxicillin and tetracycline could be used as a proxy of MDR in *E. coli* isolates from animals. As the concordance between co-resistance to amoxicillin and tetracycline and MDR is not perfect, this indicator could only be used to approximate MDR proportion.

**TABLE 4:** Concordance between multidrug resistance and co-resistance to amoxicillin and tetracycline in *E. coli* isolates per animal species, over the 2012–2016 period.

**Animal species**	**Number of isolates with co-resistance to amoxicillin and tetracycline**	**Number of multidrug resistant isolates**	**Number of multidrug resistant isolates with co-resistance to amoxicillin and tetracycline**	**Proportion of isolates with co-resistance to amoxicillin and tetracycline and also multidrug resistant**	**Proportion of isolates with co-resistance to amoxicillin and tetracycline but not multidrug resistant**	**Kappa value**
Cattle	13,936	12,021	11,137	79.9%	20.1%	0.64
Horse	394	489	382	97.0%	3.0%	0.83
Dog	650	752	572	88.0%	12.0%	0.77
Swine	2,771	2,664	2,386	86.1%	13.9%	0.70
Poultry	4,178	3,596	3,233	77.4%	22.6%	0.78
Turkey	1,507	1,023	983	65.2%	34.8%	0.68
Duck	1,291	1,132	1,039	80.5%	19.5%	0.75

To constitute a relevant proxy for MDR, the indicator should be a feasible and convenient. In animal health, resistances to amoxicillin and tetracycline are routinely tested by veterinary laboratories (Boireau et al., 2018a) because these antibiotics are among the most frequently used in veterinary medicine (Anses-ANMV, 2018). Finally, because it can be routinely monitored, co-resistance to amoxicillin and tetracycline is both an efficient and relevant indicator of MDR in *E. coli* isolated from animals. Further studies should now evaluate if this indicator remains valid for other bacterial species and in other contexts of antibiotic use. In addition, to confirm and generalize the relevance of co-resistance to amoxicillin and tetracycline as an indicator of MDR in *E. coli* isolates from animals, similar studies performed in other countries and geographical regions are needed.

Moreover, co-resistance to amoxicillin and tetracycline presents several advantages as an indicator. This co-resistance is based on a resistance profile to two pre-defined antibiotics and, consequently, is not related to the total number of antibiotic categories selected for the analysis, contrary to MDR. Using co-resistance to amoxicillin and tetracycline will avoid having to take into account a specific number of antibiotic categories for comparison. Since its definition is straightforward (providing no latitude for interpretation), co-resistance to amoxicillin and tetracycline is also easy to calculate.

## Conclusion

Beyond the conventional MDR indicator, monitoring resistance associations in animal health would greatly benefit from an easy-to-test indicator of MDR. Because it is based on a specific resistance profile and not on an arbitrary number of resistances and given the widespread use of penicillins and tetracyclines, co-resistance to tetracycline and amoxicillin might be a convenient and reliable indicator to approximate MDR in *E. coli* isolated from animals. This co-resistance could be routinely monitored to characterize the evolution of resistance’ associations over time in *E. coli* isolates. Any observed changes could then be placed in perspective in terms of therapeutic options in veterinary medicine and public health issues.

## Data Availability Statement

The data used for this study was obtained from the RESAPATH network and the access to data is controlled by the French Agency for Food, Environmental and Occupational Health & Safety (ANSES). Conditions of approval (respecting the anonymity of farms and laboratories) do not allow us to distribute or make available data directly to other parties.

## Author Contributions

All authors listed have made a substantial, direct and intellectual contribution to the work, and approved it for publication.

## Conflict of Interest

The authors declare that the research was conducted in the absence of any commercial or financial relationships that could be construed as a potential conflict of interest.

## Footnotes

1 www.sfm-microbiologie.org/
