# Supplementary material for: Co-resistance to Amoxicillin and Tetracycline as an Indicator of Multidrug Resistance in Escherichia coli Isolates From Animals
Source: Front Microbiol. 2019 Oct 9;10:2288. doi: 10.3389/fmicb.2019.02288 (PMC6794424; doi:10.3389/fmicb.2019.02288)
Supplement: Supplementary file 1 [file Table_1.DOCX]

**Supplementary Table 1.** Proportions (%[CI]) of isolates with the co-resistance amoxicillin-tetracycline plus another resistance, for all animal species considered, over the 2012-2016 period.

| Combination of resistance | Proportion of isolates with only these three resistances | Proportion of isolates including these three resistances |
| --- | --- | --- |
| AMX-TET- FLUO | 0.7 [0.7; 0.8] | 9.5 [9.3; 9.8] |
| AMX-TET-CEF | 0.7 [0.7; 0.8] | 4.5 [4.3; 4.7] |
| AMX-TET-AMC | 7.7 [7.5; 7.9] | 21.7 [21.4; 22.1] |
| AMX-TET-SXT | 7.5 [7.3; 7.7] | 22.6 [22.3; 23.0] |
| AMX-TET- GEN | 0.3 [0.3; 0.4] | 7.7 [7.5; 8.0] |

Legend: AMX: amoxicillin; FLUO: fluoroquinolones; CEF: ceftiofur; AMC: Amoxicillin and clavulanic acid; SXT: Trimethoprim-sulfamethoxazole; TET: tetracycline; GEN: gentamicin
