# Supplementary material for: Co-resistance to Amoxicillin and Tetracycline as an Indicator of Multidrug Resistance in Escherichia coli Isolates From Animals
Source: Front Microbiol. 2019 Oct 9;10:2288. doi: 10.3389/fmicb.2019.02288 (PMC6794424; doi:10.3389/fmicb.2019.02288)
Supplement: Supplementary file 2 [file Table_2.DOCX]

**Supplementary Table 2.** Matrix of correlations between multidrug resistance (MDR) and different binary combinations of resistance, all including resistance to amoxicillin.

|  | MDR | AMX-GEN | AMX-CEF | AMX-FLUO | AMX-SXT | AMX-AMC | AMX-TET |
| --- | --- | --- | --- | --- | --- | --- | --- |
| MDR | 1.00 | 0.38 | 0.29 | 0.42 | 0.66 | 0.63 | 0.75 |
| AMX-GEN | 0.38 | 1.00 | 0.20 | 0.36 | 0.36 | 0.28 | 0.29 |
| AMX-CEF | 0.29 | 0.20 | 1.00 | 0.18 | 0.16 | 0.19 | 0.20 |
| AMX-FLUO | 0.42 | 0.36 | 0.18 | 1.00 | 0.34 | 0.30 | 0.32 |
| AMX-SXT | 0.66 | 0.36 | 0.16 | 0.34 | 1.00 | 0.28 | 0.48 |
| AMX-AMC | 0.63 | 0.28 | 0.19 | 0.30 | 0.28 | 1.00 | 0.45 |
| AMX-TET | 0.75 | 0.29 | 0.20 | 0.32 | 0.48 | 0.45 | 1.00 |

Legend: MDR: Multidrug resistance, AMX: amoxicillin; GEN: gentamicin; CEF: ceftiofur; FLUO: fluoroquinolones; SXT: Trimethoprim-sulfamethoxazole; AMC: Amoxicillin and clavulanic acid; TET: tetracycline
